# Estimating the generation time for SARS-CoV-2 transmission using United States household data, December 2021–May 2023

**DOI:** 10.1038/s41598-026-46596-6

**Published:** 2026-04-11

**Authors:** Louis Yat Hin Chan, Sinead E. Morris, Melissa S. Stockwell, Natalie M. Bowman, Edwin Asturias, Suchitra Rao, Karen Lutrick, Katherine D. Ellingson, Huong Q. Nguyen, Yvonne Maldonado, Son H. McLaren, Ellen Sano, Jessica E. Biddle, Sarah E. Smith-Jeffcoat, Matthew Biggerstaff, Melissa A. Rolfes, H. Keipp Talbot, Carlos G. Grijalva, Rebecca K. Borchering, Alexandra M. Mellis, The RVTN-Sentinel Study Group, Melissa S. Stockwell, Natalie M. Bowman, Edwin Asturias, Suchitra Rao, Karen Lutrick, Katherine D. Ellingson, Huong Q. Nguyen, Yvonne Maldonado, Son H. McLaren, Ellen Sano, Jessica E. Biddle, Sarah E. Smith-Jeffcoat, Melissa A. Rolfes, Carlos G. Grijalva, Alexandra M. Mellis, H. Keipp Talbot, Lisa Saiman, Raul A. Silverio Francisco, Anny L.Diaz Perez, Ana M. Valdez de Romero, Ayla Bullock, Amy Yang, Quenla Haehnel, Jessica Lin, Julienne Reynolds, Katherine Katie Murray, Miriana Moreno Zivanovich, Anna McShea, Brittney Figueroa, Melody Liu, Kathleen Grice, Cameron Bendalin, Sonia Chavez, Jolie Granger, Ferris Alaa Ramadan, Flavia Maria Nakayima Miiro, Josue Ortiz, Mokenge Ndiva Mongoh, Edward A. Belongia, Hannah Berger, Vicki Moon, Gina Burbey, Leila Deering, Brianna Freund, Garrett Heuer, Sarah Kopitzke, Carrie Marcis, Jennifer Meece, Jennifer Moran, DeeAnn Hertel, Joshua Petrie, Miriah Rotar, Carla Rottscheit, Elisha Stefanski, Sandy Strey, Melissa Strupp, Rosita Thiessen, Marcela Lopez, Alondra A. Aguilar, Emma Stainton, Grace K-Y. Tam, Jonathan Altamirano, Leanne X. Chun, Rasika Behl, Samantha A. Ferguson, Yuan J. Carrington, Frank S. Zhou, Chris Lindsell, Judy King, John Meghreblian, Samuel Massion, Brittany Creasman, Lauren Milner, Andrea Stafford Hintz, Jorge Celedonio, Ryan Dalforno, Maria Catalina Padilla-Azain, Daniel Chandler, Paige Yates, Brianna Schibley-Laird, Alexis Perry, Ruby Swaimn, Mason Speirs, Erica Anderson, Suryakala Sarilla, Amelia Dodds, Dayton Marchlewski, Timothy Williams, Afan Swan, Onika Abrams, Jackson Resser, Ine Sohn, Cara Lwin, Hsi-nien Jubilee Tan, Stephen Yeargin, James Grindstaff, Heather Prigmore, Jessica Lai, Zhouwen Liu, James D. Chappell, Marcia Blair, Rendie E. McHenry, Bryan P. M. Peterson, Lauren J. Ezzell

**Affiliations:** 1 Centers for Disease Control and Prevention, 1600 Clifton Road, Atlanta, GA 30329 USA; 2 Columbia University Irving Medical Center, New York, NY USA; 3 University of North Carolina at Chapel Hill, Chapel Hill, NC USA; 4 University of Colorado School of Medicine and Children’s Hospital Colorado, Aurora, CO USA; 5 University of Arizona, Tucson, AZ USA; 6 Marshfield Clinic Research Institute, Marshfield, WI USA; 7 Stanford University, Stanford, CA USA; 8 Vanderbilt University Medical Center, Nashville, TN USA; 9 Goldbelt Professional Services, Chesapeake, VA USA

**Keywords:** Epidemiology, Epidemiology

## Abstract

Generation time, representing the interval between infection events in primary and secondary cases, is important for understanding disease transmission dynamics including predicting the effective reproduction number (Rt), which informs public health decisions. While previous estimates of SARS-CoV-2 generation times have been reported for early Omicron variants, there is a lack of data for subsequent sub-variants, such as XBB. We estimated SARS-CoV-2 generation times using data from the Respiratory Virus Transmission Network – Sentinel (RVTN-S) household transmission study conducted across seven U.S. sites from December 2021 to May 2023. The study spanned three Omicron sub-periods dominated by the sub-variants BA.1/2, BA.4/5, and XBB. We employed a Susceptible-Exposed-Infectious-Recovered (SEIR) model with a Bayesian data augmentation method that imputes unobserved infection times of cases to estimate the generation time. The estimated mean generation time for the overall Omicron period was 3.5 days (95% credible interval, CrI: 3.3–3.7). During the sub-periods, the estimated mean generation times were 3.8 days (95% CrI: 3.4–4.2) for BA.1/2, 3.5 days (95% CrI: 3.3–3.8) for BA.4/5, and 3.5 days (95% CrI: 3.1–3.9) for XBB. Our study provides estimates of generation times for the Omicron variant, including the sub-variants BA.1/2, BA.4/5, and XBB. These up-to-date estimates specifically address the gap in knowledge regarding these sub-variants and are consistent with earlier studies. They enhance our understanding of SARS-CoV-2 transmission dynamics by aiding in the prediction of Rt, offering insights for improving COVID-19 modeling and public health strategies.

**Supplementary Information:**

The online version contains supplementary material available at 10.1038/s41598-026-46596-6.

## Introduction

The generation time is a fundamental epidemiological concept for understanding infectious disease transmission dynamics and represents the time between beginning of infection events in primary and secondary cases. Accurate generation time estimation is important for predicting the effective reproduction number (Rt)^1,2^, which helps inform situational awareness and public health decisions. Ideally, Rt estimation relies on infection times and the generation time distribution, but because infection times are rarely observed, generation time is often inferred from the serial interval, the time between symptom onset in primary and secondary cases, which can introduce biases.

Previous estimates of SARS-CoV-2 generation times^3–6^ have been reported using data through the early SARS-CoV-2 Omicron variant period. For example, Park et al.^3^ analyzed data from the Netherlands and found that Omicron variant had a shorter mean generation time than Delta variant, emphasizing the need for variant-specific estimates. Similarly, Mefsin et al.^4^ reported that the Omicron sub-variant BA.2 in Hong Kong was associated with a shorter generation time and serial interval compared to earlier variants. However, there is a lack of estimates for more recent SARS-CoV-2 Omicron variant periods of dominance (e.g., sub-variant XBB), despite evidence showing that generation times have substantially decreased over the course of the pandemic, particularly across the Alpha, Delta, and Omicron variants^7^. This trend has corresponded to substantial increases in population immunity, and the emergence of distinct Omicron sub-variants, which may have impacted the generation time of SARS-CoV-2^8^.

Critically, shorter generation times compress transmission windows, reducing the utility of post-symptomatic mitigation measures like isolation and elevating the importance of pre-symptomatic transmission interception. A substantial proportion of SARS-CoV-2 transmission occurs prior to symptom onset, limiting the effectiveness of interventions that rely solely on isolating symptomatic cases. Therefore, understanding the distribution of generation times directly impacts the design and timing of mitigation strategies.

Updating variant-specific generation time estimates are needed to mitigate biases in Rt calculations, improve real-time assessment of epidemic trends, and prevent misinformed policy decisions. Furthermore, as the interplay between viral evolution, population immunity, and behavioral changes continues to shape transmission dynamics, contemporary estimates are essential for accurate modeling and forecasting.

To our knowledge, no previous study has provided U.S.-specific estimates covering the later Omicron sub-variant, XBB. In this study, we provide updated estimates of the generation time for more recent SARS-CoV-2 Omicron variant periods, specifically the sub-variants BA.1/2, BA.4/5, and XBB, using data from a U.S. household transmission study conducted from December 2021 to May 2023. Estimating the generation time is methodologically challenging, particularly due to the difficulty of pinpointing infection events. We employ a mechanistic Susceptible-Exposed-Infectious-Recovered (SEIR) model with Bayesian data augmentation, based on a previously published method^9^, to impute unobserved infection times and estimate both intrinsic and realized household generation times. Our findings aim to inform more accurate real-time Rt estimation, enhance epidemic modeling, and guide public health strategies in the context of evolving SARS-CoV-2 transmission dynamics.

## Methods

### Household data

Participants were enrolled in a case-ascertained household transmission study, called the Respiratory Virus Transmission Network – Sentinel (RVTN-S), from seven sites across the U.S., 2021–2023, as described in prior reports^10–13^. A convenience sample of non-hospitalized individuals diagnosed with COVID-19 were enrolled as index cases from testing at outpatient medical centers, health systems, or through ongoing surveillance or public health registries. Households were then screened by study staff via phone, and enrolled if the index case was the first person in the household who was ill, and if enrollment could occur within 7 days of the initial illness onset within the household. Households were only enrolled if at least two-thirds of all household contacts, defined as persons who routinely slept in the same household and planned to do so during the study, consented to participate. In addition, at least one household member was required to report not being ill on the date of the earliest illness onset in the household. Full inclusion and exclusion criteria are available in the study protocol^14^.

At enrollment, participants including index cases and household contacts self-reported demographics, history of SARS-CoV-2 infection, and vaccination status and provided an acute blood specimen via Asante dried blood specimen collection strips or Neoteryx Mitra cartridge devices. Participants were then monitored prospectively for 10 days, and during this follow-up period participants completed electronic diaries in which they reported daily symptoms, including fever (including feeling feverish and chills), cough, sore throat, runny nose, nasal congestion, fatigue (including feeling run-down), wheezing, trouble breathing (including shortness of breath), chest tightness (including chest pain), loss of smell or loss of taste, headache, abdominal pain, diarrhea, vomiting, and muscle or body aches. Daily nasal swabs were also collected and tested for SARS-CoV-2 via RT-PCR. Blood specimens were tested using the Thermo ProcartaPlex assay on the Luminex MAGPIX platform, and median fluorescence intensity values were used to identify serologic evidence of prior infection^12^. Individuals were considered vaccinated based on plausible self-report (dose plus location and date) or records review; individuals were considered to have a prior infection based on plausible self-report of a prior positive test (with date) or serologic evidence of anti-nucleocapsid antibodies at baseline.

### Estimating the generation time

We employed a mechanistic SEIR compartmental model and used a Bayesian Markov chain Monte Carlo (MCMC) approach for parameter estimation and data augmentation, as proposed by Hart et al.^9^. The SEIR model assumes homogeneous mixing within households, meaning all household members have an equal probability of contact and transmission. This simplification is reasonable for household settings with frequent close interactions. The model also assumes no external infections after study enrollment, attributing all secondary cases to the primary household case. This reflects the short follow-up period and the low probability of external exposure.

The SEIR model incorporates three infectious compartments: asymptomatic, pre-symptomatic, and symptomatic stages. After infection, individuals enter a non-infectious exposed phase before progressing to an infectious state. This structure explicitly accounts for the latent period between exposure and infectiousness, which is a critical feature for SARS-CoV-2 given its significant pre-symptomatic transmission. Individuals then follow through one of two pathways: either remaining asymptomatic or developing symptoms following a pre-symptomatic phase. Consequently, transmission can occur before the onset of symptoms, depending on the length of the incubation period. We assumed the incubation period had a mean of 2.6 days and a standard deviation (SD) of 1.0 days for the Omicron variant^15^. We also conducted a sensitivity analysis by assuming a longer incubation period with a mean of 4.1 days and an SD of 2.7 days^3^.

The data augmentation MCMC approach, based on the methodology from a household transmission modeling study by Cauchemez et al.^16^, was also applied in previous studies^17,18^. The detailed descriptions are provided in the Supplementary Material. We imputed symptom onsets and infection times of cases to estimate both intrinsic and realized household generation times. The intrinsic generation time assumes no depletion of susceptible individuals, reflecting infection in the community with an unlimited supply of susceptible individuals. In contrast, the realized household generation time accounts for a gradual reduction in the number of susceptible individuals as people are infected within the household setting. We also estimated the overall infectiousness, which describes the expected number of household transmissions generated by a single symptomatic infected primary case.

We examined posterior distributions through convergence diagnostics to ensure well mixing and stability of the chains. They were also compared with prior distributions to confirm parameter identifiability. Estimates are presented as posterior means with 95% credible intervals (CrI), defined by the 2.5th and 97.5th percentiles of the MCMC samples, representing the associated uncertainty.

For each sub-period relative to the overall Omicron variant period, we assessed whether CrIs overlapped. Overlapping 95% CrIs were interpreted as reflecting no statistically significant differences between estimates. To provide a more quantitative assessment, we also calculated the overlapping index, which measures posterior distribution similarity^19,20^. In Bayesian inference, where uncertainty is interpreted probabilistically as a density distribution rather than a single interval, the overlapping index quantifies the proportion of shared density between two distributions and captures differences in distributional shape and density, rather than just the range of uncertainty. Values over 95% indicate high similarity with minimal differences, while values less than 5% suggest low similarity with significant differences. For example, an overlapping index of 50% suggests moderate similarity but also meaningful differences in the distributions between sub-periods. This metric provides a distribution-based measure of similarity that complements traditional hypothesis testing (e.g., t-tests) and is particularly useful in a Bayesian framework.

The estimation was performed in R (version 4.3.1) with 1,000,000 MCMC iterations, discarding the initial 20% as burn-in and obtaining posterior distributions by thinning every 100 iterations. Each MCMC chain took approximately 48 h to run on a single core of a high-performance computing cluster. The code for the estimations is available at https://github.com/CDCgov/covid-generation_time-us.

## Results

### Household data

We enrolled 745 households with a single primary case (i.e., only one individual within a household exhibited symptoms on the earliest onset date) with laboratory-confirmed SARS-CoV-2, along with their 1,334 household contacts, totaling 2,079 individuals including both primary cases and household contacts. The study period spanned from December 2021 to May 2023, encompassing the period when the Omicron variant predominated in the U.S. This period was further categorized into three sub-periods dominated by the sub-variants BA.1/2 (December 18, 2021 – June 17, 2022), BA.4/5 (June 18, 2022 – January 14, 2023), and XBB (January 15, 2023 – May 1, 2023)^21,22^.

Of 2,079 participants, all of whom had at least two valid SARS-CoV-2 PCR tests^23^, 69.4% (95% confidence interval, CI: 67.4%–71.4%) tested positive and reported symptoms (symptomatic infected), 8.5% (95% CI: 7.4%–9.8%) tested positive but never reported symptoms (asymptomatic infected), and 22.1% (95% CI: 20.3%–23.9%) tested negative (uninfected) regardless of symptoms (Table 1). Over the study period, the proportion who remained uninfected increased from 18.5% (95% CI: 15.5–21.8) during the BA.1/2 sub-period to 29.0% (95% CI: 24.9%–33.6%) during the XBB sub-period. The 95% CIs (distinct from the 95% CrIs reported for posterior means in following sections) were calculated using the Wilson score method^24^ and the detailed descriptions are provided in the Supplementary Material.

**Table 1:** Participant symptom and infection status by SARS-CoV-2 variant periods: All Omicron (December 2021 – May 2023), and sub-variants BA.1/2 (December 18, 2021 – June 17, 2022), BA.4/5 (June 18, 2022 – January 14, 2023), and XBB (January 15, 2023 – May 1, 2023).

Period	Number of participants (households)	Symptomatic infected % (95% CI, n/N)	Asymptomatic infected % (95% CI, n/N)	Uninfected % (95% CI, n/N)
All Omicron	2079 (745)	69.4 (67.4–71.4, 1443/2079)	8.5 (7.4–9.8, 177/2079)	22.1 (20.3–23.9, 459/2079)
BA.1/2	569 (200)	72.9 (69.1–76.4, 415/569)	8.6 (6.6–11.2, 49/569)	18.5 (15.5–21.8, 105/569)
BA.4/5	1090 (389)	69.4 (66.6–72.0, 756/1090)	9.4 (7.8–11.2, 102/1090)	21.3 (19.0–23.8, 232/1090)
XBB	420 (156)	64.8 (60.1–69.2, 272/420)	6.2 (4.3–8.9, 26/420)	29.0 (24.9–33.6, 122/420)

The 95% confidence intervals (CIs) were calculated using the Wilson score method^24^.

### The generation time for the overall Omicron variant period and sub-variant periods

**Fig. 1:**
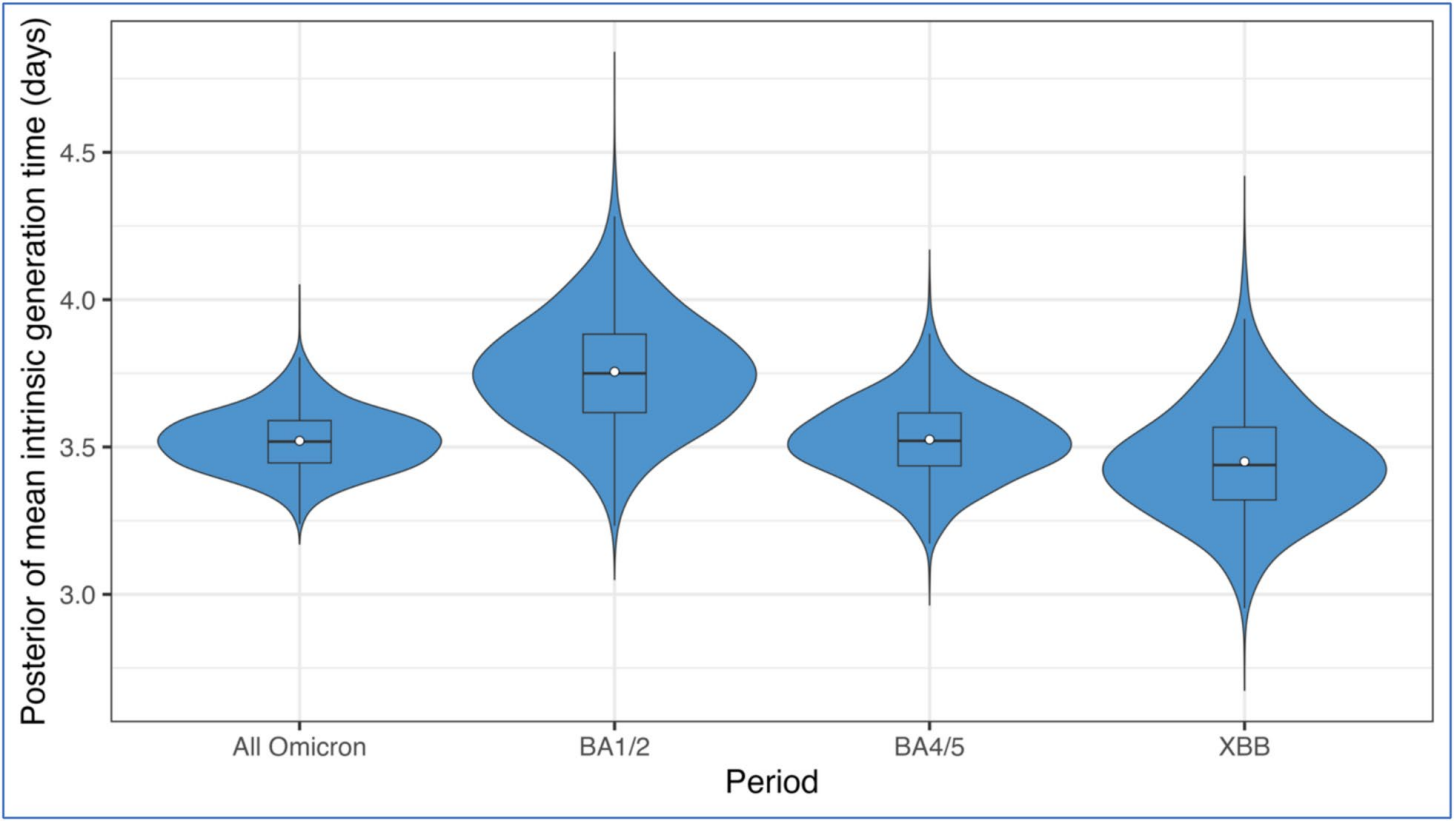
Posterior distribution of mean intrinsic generation time (assuming no depletion of susceptible individuals to reflect infection in the community with an unlimited supply of susceptible individuals) categorized by SARS-CoV-2 variant periods: Omicron (December 2021 – May 2023), and sub-variants BA.1/2 (December 18, 2021 – June 17, 2022), BA.4/5 (June 18, 2022 – January 14, 2023), and XBB (January 15, 2023 – May 1, 2023). The blue violin areas represent the kernel densities, with the width proportional to the posterior density. The superimposed box plots show the median values and interquartile ranges of the same posterior distributions. The white dots show the mean values. Each posterior distribution was summarized using 8,000 MCMC samples after burn-in and thinning. The overlapping index quantifies the similarity between each sub-period and the overall Omicron posterior distribution: BA.1/2 = 41%, BA.4/5 = 85%, and XBB = 65%.

For the overall Omicron variant period, we found a mean intrinsic generation time of 3.5 days (95% CrI: 3.3–3.7) and a mean realized household generation time of 3.0 days (95% CrI: 2.8–3.1) (Table 2 and Fig. 1). The estimates during the sub-periods were similar to the overall Omicron estimate, with overlapping 95% CrIs indicating no statistically significant differences and overlapping indices above 40% reflecting substantial similarity in the underlying distributions. The overlapping indices capture differences in distributional shape by quantifying the proportion of shared density between posterior distributions, rather than relying solely on the range of uncertainty reflected in the 95% CrIs.

**Table 2:** Posterior mean (95% CrI) of estimates categorized by SARS-CoV-2 variant periods: Omicron (December 2021 – May 2023), and sub-variants BA.1/2 (December 18, 2021 – June 17, 2022), BA.4/5 (June 18, 2022 – January 14, 2023), and XBB (January 15, 2023 – May 1, 2023).

	All Omicron	BA.1/2	BA.4/5	XBB
Mean intrinsic generation time (days)	3.5 (3.3–3.7)	3.8 (3.4–4.2)	3.5 (3.3–3.8)	3.5 (3.1–3.9)
SD of intrinsic generation time (days)	2.1 (1.9–2.3)	2.2 (1.8–2.7)	2.1 (1.8–2.4)	1.8 (1.4–2.2)
Mean realized household generation time (days)	3.0 (2.8–3.1)	3.1 (2.9–3.4)	2.9 (2.7–3.2)	3.1 (2.8–3.4)
SD of realized household generation time (days)	1.8 (1.6–1.9)	1.8 (1.5–2.0)	1.8 (1.6–2.0)	1.5 (1.3–1.8)
Mean serial interval (days)	3.4 (3.2–3.6)	3.6 (3.3–4.1)	3.4 (3.1–3.7)	3.3 (2.9–3.7)
SD of serial interval (days)	2.2 (2.0–2.4)	2.3 (2.0–2.8)	2.3 (2.0–2.6)	1.9 (1.5–2.4)

We assumed the incubation period had a mean of 2.6 days and a standard deviation (SD) of 1.0 days^15^.

The mean intrinsic generation time for the BA.1/2 sub-period was slightly longer at 3.8 days (95% CrI: 3.4–4.2) (Table 2). The mean intrinsic generation time for the BA.4/5 and XBB sub-periods was 3.5 days (BA.4/5: 95% CrI: 3.3–3.8; XBB: 95% CrI: 3.1–3.9). The mean realized household generation time for each variant sub-period ranged between 2.9 and 3.1 days (BA.1/2: 3.1 days, 95% CrI: 2.9–3.4; BA.4/5: 2.9 days, 95% CrI: 2.7–3.2; XBB: 3.1 days, 95% CrI: 2.8–3.4). The standard deviations (SDs) of these estimates were consistent across the sub-periods, with the XBB sub-period exhibited marginally lower variability. Notably, the mean serial interval estimates were slightly shorter than the generation time estimates but with higher SDs (Table 2).

In the sensitivity analysis assuming the longer incubation period, we found a mean intrinsic generation time of 3.6 days (95% CrI: 3.4–3.9), with an overlapping index of 70% relative to the main analysis, demonstrating insensitivity to changes in incubation period assumptions. However, the SDs of the intrinsic generation time increased significantly (Table S2).

### The overall infectiousness

**Fig. 2:**
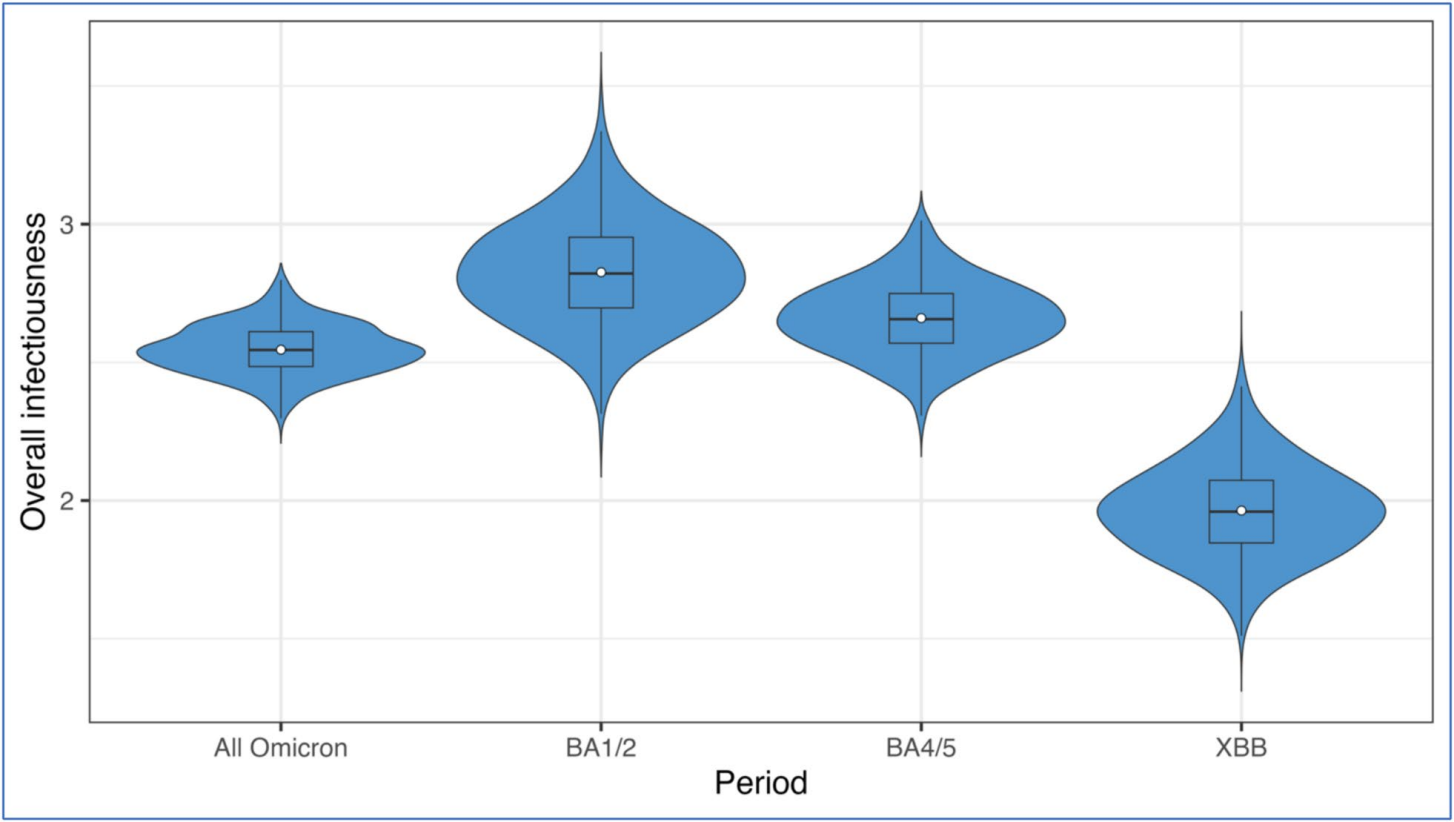
Posterior distribution of overall infectiousness (expected number of household transmissions generated by a single symptomatic infected primary case) categorized by SARS-CoV-2 variant periods: Omicron (December 2021 – May 2023), and sub-variants BA.1/2 (December 18, 2021 – June 17, 2022), BA.4/5 (June 18, 2022 – January 14, 2023), and XBB (January 15, 2023 – May 1, 2023). The blue violin areas represent the kernel densities, with the width proportional to the posterior density. The superimposed box plots show the median values and interquartile ranges of the same posterior distributions. The white dots show the mean values. Each posterior distribution was summarized using 8,000 MCMC samples after burn-in and thinning. The overlapping index quantifies the similarity between each sub-period and the overall Omicron posterior distribution: BA.1/2 = 29%, BA.4/5 = 61%, and XBB = 3%.

The overall infectiousness during the overall Omicron period was 2.5 (95% CrI: 2.4–2.7). This means that, on average, each symptomatic primary case was responsible for approximately 2.5 secondary infections within the household. Additionally, we observed a substantial decrease across each sub-period, starting at 2.8 (95% CrI: 2.5–3.2) during the BA.1/2 sub-period, followed by a slight decline to 2.7 (95% CrI: 2.4–2.9) with 61% overlapping during the BA.4/5 sub-period, and then a significant drop to 2.0 (95% CrI: 1.6–2.3) with only 3% overlapping, suggesting a statistically significant reduction during the XBB sub-period (Fig. 2 and Table S1).

## Discussion

Our estimated mean intrinsic generation time for the overall Omicron variant period was 3.5 (95% CrI: 3.3–3.7) days, which is shorter than the estimates for earlier periods of the Delta (4.7 days, 95% CrI: 4.1–5.6), Alpha (5.5 days, 95% CrI: 4.7–6.5) and wild-type variants (5.9 days, 95% CrI: 5.2–7.0)^9,18^. Our results align with a previous systematic review and meta-analysis^7^ that demonstrated a decrease in the generation time (and incubation period) across the Alpha, Delta, and Omicron variants from 2021 to 2023.

This decreasing trend in the generation time may be influenced by a combination of factors. These factors can both shorten the generation time and lower the overall infectiousness, as observed across different Omicron sub-variant periods, partly because fewer transmissions occur in the later stages of the infectious period. These include (1) less infectious infectors due to biological changes in the pathogen, such as specific virological characteristics of the variants/sub-variants, altered viral shedding patterns, or reduced viral load that lowers virus transmissibility; (2) less susceptible contacts due to higher population immunity resulting in fewer successful transmissions; and (3) behavioral changes, such as reduced direct contact with household members and more routine contact with others due to the relaxation of pandemic mitigation measures^8^. However, the exact underlying reasons for this trend remain uncertain.

The shorter generation time suggests transmission occurred earlier, implying faster transmission dynamics, which has implications for public health measures, such as the need for timely case identification and rapid implementation of control measures. If it was primarily driven by virologic factors or altered viral shedding patterns, isolation periods might be shortened and focused more intensively on the early days. Importantly, we observed a decrease in the overall infectiousness between the Omicron sub-variant periods. Despite the principle that a shorter generation time does not necessarily correlate with higher overall infectiousness, the combination of a shorter generation time paired with reduced infectiousness suggests that transmission might have occurred faster during the XBB sub-period compared to the earlier Omicron sub-periods, although the overall number of transmission events was lower.

Nevertheless, the absence of statistically significant differences in generation times among sub-variants, combined with the high overlapping indices, suggests that, despite ongoing viral evolution, the transmission dynamics within the Omicron period have remained broadly stable. This finding supports the continued use of uniform prevention guidance across sub-variants rather than tailoring measures to each new lineage. These results also highlight the importance of regularly updating and integrating empirically derived estimates into real-time Rt estimation systems so that surveillance and forecasting models remain aligned with current transmission patterns. At the same time, the marked reduction in generation time compared to earlier variants reinforces the importance of rapid detection and staying home when sick to effectively interrupt transmission.

Our results directly relate to the importance of updating generation time parameters in real-time models, such as those used to estimate Rt and forecast epidemic trends. Our intrinsic generation time estimates exceed a previous estimate of 2.9 days from a Netherlands study^3^, which is currently used to predict Rt and to infer the current epidemic growth status for states and territories in the U.S.^1^. Other current efforts, including those using R packages such as EpiNow2 and epinowcast, to forecast or model COVID-19 in the United States may rely on earlier estimates as well as estimates from other countries, which could introduce biases. For example, given historical surveillance data, an underestimated generation time would overestimate Rt and prediction trends. Updating generation time estimates to reflect more recent data and current community transmission settings could improve the accuracy of real-time infection trend predictions^2^. In addition, it is important to consider the range of uncertainty in these estimates when utilizing them for Rt estimation. Incorporating the full posterior distribution of the generation time parameter in the Rt estimation procedure ensures that uncertainty in the generation time is properly reflected in Rt calculations^2^, leading to more reliable and interpretable epidemic trend estimates. Integrating our estimated generation time distribution into existing systems is straightforward, as these platforms typically allow user-defined generation time kernels.

Our sensitivity analysis showed that assuming a longer incubation period did not substantially change the mean intrinsic generation time but increased the variability. This is consistent with the expectation that a longer and more variable incubation period introduces greater uncertainty when imputing infection times from symptom onset. While the average transmission timing remains robust, precise incubation period characterization is critical for reducing uncertainty in generation time estimates.

Our estimated realized household generation time for the overall Omicron variant period was 3.0 days (95% credible interval, CrI: 2.8–3.1), half a day shorter than the intrinsic generation time. This difference arises because finite household sizes lead to rapid depletion of susceptible individuals, unlike the assumption of unlimited susceptible individuals used in the intrinsic generation time. Similar estimates were found in earlier studies^3–5^, and a pooled mean for Omicron BA.1 of 3.0 days (95% confidence interval, CI: 2.5–3.5)^7^ closely matches our estimates for the BA.1/2 sub-period of 3.1 days (95% CrI: 2.9–3.4). Furthermore, Wang et al.^6^ found that the mean realized household generation time of Omicron BA.5 variants was 2.8 days (95% CrI: 2.4–3.5), also closely matching our estimate for the BA.4/5 sub-period of 2.9 days (95% CrI: 2.7–3.2). This consistency confirms the robustness of our estimates.

Our study has several limitations. First, we estimated the generation time across the entire study population, regardless of prior immunity from vaccination or previous infection, symptomatic status, or age. Our primary objective was to provide more up-to-date population-level estimates for Rt estimation^2^, which reflect the combined effects of shifting population immunity, behavioral changes, and evolving viral lineages, rather than to estimate specific generation times by immune status or age group. While previous research^18^ has shown that fully vaccinated individuals may experience slightly longer generation times, our supplementary analysis was consistent with this finding. We stratified households by vaccination and prior infection status (comparing households where all members were vaccinated or previously infected to those where all were unvaccinated or previously uninfected). We found that households with everyone vaccinated or previously infected had a longer mean generation time, but the difference was not statistically significant (Supplementary Material). However, it is important to note that the majority of study participants were vaccinated, with fewer than 10% of households consisting entirely of unvaccinated individuals, resulting in estimates with wide credible intervals and limited power for subgroup comparisons. Because households were recruited primarily through healthcare and surveillance networks, our sample likely overrepresented healthcare-seeking individuals and underrepresented those with asymptomatic infection. Similarly, this study only enrolled individuals who continued to reside in households during their illness period, limiting our ability to describe the full spectrum of illness severity, including among hospitalized cases. These estimates should therefore be interpreted as exploratory and descriptive rather than inferential, and our sample may not be fully representative of the general population.

Second, we assumed a single incubation period distribution for the entire study population, regardless of age, viral lineage, or host factors. We acknowledge that the true incubation period likely varies by individual characteristics. However, modeling group-specific variations in incubation periods is challenging because infection times are rarely observed directly and are difficult to estimate precisely. Our sensitivity analysis demonstrated that the mean generation time remained robust to an alternative incubation period distribution, although variability increased.

Third, we assumed homogeneous mixing among household members may introduce bias if behavioral heterogeneity exists (e.g., self-isolation practices reducing contact). This simplification, while common in household models, could overestimate the overall infectiousness in larger households with varied interaction patterns. Moreover, our enrollment occurred during periods of evolving public-health guidance, such as changing recommendations on masking, isolation, and quarantine, which may have altered contact patterns over time.

Fourth, we did not consider external infection routes outside the households. Household members might have been infected in the community, leading to an overestimation of the overall infectiousness. We identified 4 households with observed serial intervals greater than 10 days, which may indicate possible community transmission events. Among over 700 households, excluding these few outliers did not change the overall estimates of the generation time, indicating that our findings are robust to their inclusion. Nevertheless, we acknowledge that unmeasured community exposures cannot be fully ruled out.

Fifth, we did not consider virological parameters such as viral load or shedding duration, which limits the biological interpretability of our generation time estimates. These data were beyond the scope of the current study, but integrating virological measures in future work could enhance the biological grounding of our model. Quantitative viral load trajectories would allow direct examination of how the timing, intensity, and duration of infectiousness shape the observed distribution. This could clarify how generation time relates to both the magnitude and temporal profile of potential infectiousness. For example, viral load trajectories could help explore whether shortened generation times reflect earlier peak infectiousness or a reduced duration of viral shedding. Additionally, we were unable to empirically investigate the causal mechanisms underlying observed differences in our estimates. While we discuss potential contributors, such as changes in viral characteristics, rising population immunity, and behavioral adaptations, our analysis does not disentangle their individual or combined effects. Future studies should also prioritize age-stratified analyses, incorporate virological parameters, and continue to monitor emerging variants and update generation time estimates to inform real-time modeling and public health responses. In addition, advanced analytical methods, including machine learning and deep learning frameworks, hold promise for uncovering complex, nonlinear relationships within epidemiological data and can enhance predictive accuracy beyond traditional compartmental models. Integrating these innovative approaches with mechanistic models may further improve our ability to anticipate and respond to evolving SARS-CoV-2 transmission dynamics.

## Conclusions

Based on SARS-CoV-2 household transmission data collected from December 2021 to May 2023 in the U.S., we present updated estimates of the intrinsic and realized household generation time. These estimates offer valuable insights into the transmission dynamics of SARS-CoV-2 within communities and households. Our results are crucial for enhancing COVID-19 modeling and public health strategies and highlight the necessity of ongoing evaluation of transmission patterns for optimal outbreak management.

## Supplementary Information

Supplementary Information.

## Data Availability

The datasets used and analyzed during the current study are available from the corresponding author on reasonable request. The R code for estimating generation time is available at https://github.com/CDCgov/covid-generation_time-us.
