# Supplementary material for: Estimating the generation time for SARS-CoV-2 transmission using United States household data, December 2021–May 2023
Source: Sci Rep. 2026 Apr 11;16:16890. doi: 10.1038/s41598-026-46596-6 (PMC13230514; doi:10.1038/s41598-026-46596-6)
Supplement: Supplementary file 1 — Supplementary Information. [file 41598_2026_46596_MOESM1_ESM.pdf]

Estimating the generation time for SARS-CoV-2 transmission using United States household data, December 2021–May 2023

Louis Yat Hin Chan[1,#], Sinead E. Morris[1,9], Melissa S. Stockwell[2], Natalie M. Bowman[3], Edwin Asturias[4], Suchitra Rao[4], Karen Lutrick[5], Katherine D. Ellingson[5], Huong Q. Nguyen[6], Yvonne Maldonado[7], Son H. McLaren[2], Ellen Sano[2], Jessica E. Biddle[1], Sarah E. Smith-Jeffcoat[1], Matthew Biggerstaff[1], Melissa A. Rolfes[1], H. Keipp Talbot[8], Carlos G. Grijalva[8], Rebecca K. Borchering[1], Alexandra M. Mellis[1] on behalf of the RVTN-Sentinel Study Group*

1. Centers for Disease Control and Prevention, Atlanta, GA, USA
2. Columbia University Irving Medical Center, New York, NY, USA
3. University of North Carolina at Chapel Hill, Chapel Hill, NC, USA
4. University of Colorado School of Medicine and Children's Hospital Colorado, Aurora, CO, USA
5. University of Arizona, Tucson, AZ, USA
6. Marshfield Clinic Research Institute, Marshfield, WI, USA
7. Stanford University, Stanford, CA, USA
8. Vanderbilt University Medical Center, Nashville, TN, USA
9. Goldbelt Professional Services, Chesapeake, VA, USA

* A list of authors and their affiliations appears at the end of the paper.

[#] Influenza Division, Centers for Disease Control and Prevention, 1600 Clifton Road, Atlanta, GA 30329, USA (LouisChan@cdc.gov)

Supplementary material

Model parameter estimation

The SEIR model consists of six compartments: susceptible (S), latent (E), asymptomatic infectious (A), pre-symptomatic infectious (P), symptomatic infectious (I), and recovered (R). After exposure, individuals first enter the latent (E) compartment, during which they are infected but not yet infectious. The durations of the latent period (y_E) and the pre-symptomatic infectious period (y_P) are both assumed to follow gamma distributions, specifically $y_E \sim Gamma\left(k_E, \frac{1}{(k_E+k_P)\gamma}\right)$ and $y_P \sim Gamma\left(k_P, \frac{1}{(k_E+k_P)\gamma}\right)$. The mean durations for these periods are $\frac{k_E}{(k_E+k_P)\gamma}$ and $\frac{k_P}{(k_E+k_P)\gamma}$, respectively. The incubation period (τ_{inc}), defined as the sum of the latent and pre-symptomatic periods, is also gamma distributed, $\tau_{inc} \sim Gamma\left(k_{inc}, \frac{1}{k_{inc}\gamma}\right)$, where $k_{inc} = k_E + k_P$ is the shape parameter and $\frac{1}{\gamma}$ is the mean incubation period. The symptomatic infectious period (y_I) is modeled with an exponential distribution, $y_I \sim Exp(\mu)$, where $\frac{1}{\mu}$ is the mean symptomatic infectious period.

The force of infection, $\beta(\tau)$, for an infected individual at time since infection τ, is defined as $\beta(\tau) = \alpha \frac{\beta_0}{n} f(\tau)$, where β_0 is the overall infectiousness (the expected number of household transmissions generated by a single symptomatic primary case), n is the household size, $f(\tau)$ is the generation time

distribution, and α is the relative infectiousness for different infectious stages. In this model, the relative infectiousness for symptomatic individuals (α_I) is set to 1, while for asymptomatic individuals (α_A), it is set to 0.35. The relative infectiousness for pre-symptomatic individuals (α_P) is estimated from the data, representing the ratio of pre-symptomatic to symptomatic transmission rates. Additional estimated parameters include the ratio of the mean latent period to the mean incubation period ($\frac{k_E}{k_{inc}}$), the mean symptomatic infectious period ($\frac{1}{\mu}$), and the overall infectiousness (β_0). The vector of parameters, $\theta = \left(\frac{k_E}{k_{inc}}, \frac{1}{\mu}, \alpha_P, \beta_0\right)$, is estimated using a Bayesian data augmentation Markov chain Monte Carlo (MCMC) method. The prior distributions for the model parameters (Table S1) were taken from the earlier study by Hart et al. (Hart, et al. 2022).

**Model parameter**	**Prior distribution / prior mean (95% CrI)**	**Posterior mean (95% CrI)**			
		**All Omicron**	**BA.1/2**	**BA.4/5**	**XBB**
Ratio of the mean latent period to the mean incubation period ($\frac{k_E}{k_{inc}}$)	Beta (2.1, 2.1) / 0.5 (0.1–0.9)	0.24 (0.03–0.55)	0.40 (0.07–0.74)	0.29 (0.07–0.59)	0.66 (0.30–0.89)
Mean symptomatic infectious period ($\frac{1}{\mu}$)	Lognormal (1.6, 0.8) / 5.0 (1.0–23.8)	1.68 (1.44–1.94)	1.92 (1.49–2.46)	1.76 (1.45–2.13)	1.48 (0.98–2.12)
Relative infectiousness for pre-symptomatic individuals (α_P)	Lognormal (0, 0.8) / 1.0 (0.2–4.8)	0.41 (0.21–0.83)	0.63 (0.24–1.52)	0.53 (0.26–1.10)	1.28 (0.29–3.89)
Overall infectiousness (β_0)	Lognormal (0.7, 0.8) / 2.0 (0.4–9.7)	2.5 (2.4–2.7)	2.8 (2.5–3.2)	2.7 (2.4–2.9)	2.0 (1.6–2.3)

Table S1. Prior distribution and the posterior mean (95% CrI) of model parameters categorized by SARS-CoV-2 variant periods: Omicron (December 2021 – May 2023), and sub-variants BA.1/2 (December 18, 2021 – June 17, 2022), BA.4/5 (June 18, 2022 – January 14, 2023), and XBB (January 15, 2023 – May 1, 2023).

The Bayesian data augmentation MCMC approach jointly estimates model parameters and augments the observed data with latent variables, specifically infection times and symptom onset times for each individual. Each iteration of the MCMC chain involves four steps:

1. New model parameters are proposed using a multivariate normal proposal distribution.
2. New symptom onset times for symptomatic individuals are proposed using independent uniform proposal distributions.
3. New infection times for symptomatic individuals are proposed using independent normal proposal distributions.
4. New infection times for asymptomatic individuals are proposed using independent normal proposal distributions.

At each step, proposed values are accepted or rejected based on the acceptance probability, calculated as the ratio of the proposed posterior to the current posterior. The posterior distribution is the product of the likelihood and the prior distribution, and for the steps involving latent variables, the acceptance probability simplifies to the ratio of the proposed likelihood to the current likelihood. If this ratio exceeds one, the proposed values are automatically accepted.

For a household of size n, the likelihood function consists of two parts: the contribution from transmission $L_{k,1}(D|\theta)$, and the incubation period $L_{k,2}(D|\theta)$ for each individual k, given parameter θ and data D. The overall likelihood is expressed as $L(D|\theta) = \prod_{k=1}^{n} L_{k,1}(D|\theta)L_{k,2}(D|\theta)$.

1. The likelihood of transmission $L_{k,1}(D|\theta)$:
   a. For a primary case k, the likelihood of transmission is set to $L_{k,1}(D|\theta) = 1$.
   b. For an infected individual k with infection time t_k, the likelihood of transmission is given by $L_{k,1}(D|\theta) = \lambda(t_k)e^{-\int_{-\infty}^{t_k}\lambda(s)ds}$, where the total force of infection is $\lambda(t_k) = \sum_j \beta(t_k - t_j)$ and t_j is the infection time of other earlier infected household members j.
   c. For an uninfected individual k, taking $t_k = +\infty$, the likelihood of transmission becomes $L_{k,1}(D|\theta) = e^{-\int_{-\infty}^{+\infty}\lambda(s)ds}$.
2. The likelihood of the incubation period $L_{k,2}(D|\theta)$:
   a. For an infected individual k, the likelihood of the incubation period is given by $L_{k,2}(D|\theta) = f_{inc}(\eta_k - t_k)$, where $f_{inc}(\eta_k - t_k)$ represents the incubation period distribution and η_k is the time of symptom onset for individual k.
   b. For an uninfected individual k, the likelihood of the incubation period is set to $L_{k,2}(D|\theta) = 1$.

Wilson confidence intervals

The 95% confidence intervals (CIs) for the proportions presented in Table 1 were calculated using the Wilson score method (Wilson 1927). This method accounts for binomial uncertainty while avoiding overshoot or zero-width intervals that can occur with standard normal approximation methods. For each period, the proportion of symptomatic infected, asymptomatic infected, and uninfected participants was treated as a binomial proportion. The 95% Wilson CIs were computed using the formula: $\frac{p+\frac{z^2}{2N}\pm z\sqrt{\frac{p(1-p)}{N}+\frac{z^2}{4N^2}}}{1+\frac{z^2}{N}}$ or equivalently, $\frac{p+\frac{t}{2}\pm\sqrt{p(1-p)t+\frac{t^2}{4}}}{1+t}$, where $p = \frac{n}{N}$, $t = \frac{z^2}{N}$, and $z = 1.96$ for a 95% confidence level. The Wilson score method produces asymmetric intervals, which is particularly evident when the observed proportion is close to zero or one. For example, for the overall Omicron variant period, the proportion of asymptomatic infected participants was 8.5% (n = 177 out of N = 2,079), with a 95% CI of 7.4% to 9.8%. The upper bound of this interval is slightly wider than the lower bound.

Estimates of generation time using a longer incubation period

	**All Omicron**	**BA.1/2**	**BA.4/5**	**XBB**
Mean intrinsic generation time (days)	3.6 (3.4–3.9)	4.0 (3.5–4.5)	3.7 (3.3–4.2)	3.9 (3.3–4.6)
SD of intrinsic generation time (days)	2.7 (2.6–2.8)	2.8 (2.6–3.0)	2.7 (2.6–2.8)	2.7 (2.6–2.9)
Mean realized household generation time (days)	2.6 (2.3–2.9)	2.9 (2.4–3.6)	2.6 (2.2–3.2)	3.0 (2.3–3.9)
SD of realized household generation time (days)	2.0 (1.8–2.1)	2.0 (1.7–2.4)	1.9 (1.7–2.2)	2.1 (1.7–2.5)
Mean serial interval (days)	2.3 (1.9–2.7)	2.9 (2.2–3.6)	2.3 (1.8–2.9)	2.5 (1.8–3.4)
SD of serial interval (days)	3.6 (3.4–3.8)	3.6 (3.2–4.0)	3.6 (3.3–3.9)	3.5 (3.1–3.9)

Table S2. Posterior mean (95% CrI) of estimates categorized by SARS-CoV-2 variant periods: Omicron (December 2021 – May 2023), and sub-variants BA.1/2 (December 18, 2021 – June 17, 2022), BA.4/5 (June 18, 2022 – January 14, 2023), and XBB (January 15, 2023 – May 1, 2023). This sensitivity analysis assumed a longer incubation period with a mean of 4.1 days and a standard deviation (SD) of 2.7 days (Park, et al. 2023), instead of a mean of 2.6 days and an SD of 1.0 days used in the main analysis (Ogata and Tanaka 2023).

Estimates of generation time by immune status

The primary analysis included 2,079 individuals from 745 households. Of these, 513 households (1,289 individuals) consisted entirely of vaccinated individuals, while 36 households (112 individuals) were entirely unvaccinated. We further stratified households by prior infection status (either entirely uninfected or all members previously infected).

We estimated the mean intrinsic generation time and overall infectiousness for these subsets across the entire Omicron period (Table S3). Households with prior infection showed a longer mean intrinsic generation time (4.2 days, 95% CrI: 3.6–5.1) and lower overall infectiousness (1.8, 95% CrI: 1.4–2.2). Among vaccinated households, the mean intrinsic generation time was 3.7 days (95% CrI: 3.5–4.0) with overall infectiousness of 2.3 (95% CrI: 2.1–2.5). Among unvaccinated households, the mean intrinsic generation time was 3.2 days (95% CrI: 2.3–4.5) with overall infectiousness of 3.2 (95% CrI: 2.3–4.1).

While both vaccination and prior infection were associated with longer intrinsic generation times and reduced overall infectiousness, these effects were not statistically significant. The overlapping 95% CrIs and small sample sizes (particularly among unvaccinated and previously infected groups) limit power to detect meaningful differences. Nevertheless, these findings align with the hypothesis that immunity (from vaccination or prior infection) may slow transmission dynamics and reduce infectiousness within households.

**Vaccination status**	**Prior infection status**	**Number of participants (households)**	**Mean intrinsic generation time (95% CrIs)**	**Overall infectiousness (95% CrIs)**
All	All	2079 (745)	3.5 (3.3–3.7)	2.5 (2.4–2.7)
All	Infected	251 (99)	4.2 (3.6–5.1)	1.8 (1.4–2.2)
All	Uninfected	832 (330)	3.4 (3.2–3.7)	3.0 (2.7–3.3)
Vaccinated	All	1289 (513)	3.7 (3.5–4.0)	2.3 (2.1–2.5)
Vaccinated	Infected	142 (62)	4.6 (3.7–5.9)	1.6 (1.2–2.2)
Vaccinated	Uninfected	626 (266)	3.6 (3.3–3.9)	2.7 (2.3–3.0)
Unvaccinated	All	112 (36)	3.2 (2.3–4.5)	3.2 (2.3–4.1)
Unvaccinated	Infected	30 (10)	4.2 (2.4–7.7)	1.7 (0.8–2.8)
Unvaccinated	Uninfected	27 (9)	4.4 (2.3–8.7)	6.0 (3.2–10.2)

Table S3. Posterior mean (95% CrIs) of the mean intrinsic generation time and overall infectiousness by vaccination and prior infection status, across the entire Omicron period. The overall infectiousness refers to the expected number of household transmissions generated by a single symptomatic infected primary case. We assumed the incubation period had a mean of 2.6 days and a standard deviation (SD) of 1.0 days (Ogata and Tanaka 2023).

References

Hart, William S, Sam Abbott, Akira Endo, Joel Hellewell, Elizabeth Miller, Nick rews, Philip K Maini, Sebastian Funk, and Robin N Thompson. 2022. "Inference of the SARS-CoV-2 generation time using UK household data." *eLife* (eLife Sciences Publications, Ltd) 11: e70767.

Ogata, Tsuyoshi, and Hideo Tanaka. 2023. "SARS-CoV-2 incubation period during the Omicron BA. 5--dominant period in Japan." *Emerging Infectious Diseases* (Centers for Disease Control and Prevention) 29 (3): 595.

Park, Sang Woo, Kaiyuan Sun, Sam Abbott, Ron Sender, Yinon M. Bar-on, Joshua S. Weitz, Sebastian Funk, et al. 2023. "Inferring the differences in incubation-period and generation-interval distributions of the Delta and Omicron variants of SARS-CoV-2." *Proceedings of the National Academy of Sciences* (National Acad Sciences) 120 (22): e2221887120.

Wilson, Edwin B. 1927. "Probable inference, the law of succession, and statistical inference." *Journal of the American Statistical Association* (Taylor & Francis) 22 (158): 209--212.

RVTN-Sentinel Study Group

Melissa S. Stockwell[2], Natalie M. Bowman[3], Edwin Asturias[4], Suchitra Rao[4], Karen Lutrick[5], Katherine D. Ellingson[5], Huong Q. Nguyen[6], Yvonne Maldonado[7], Son H. McLaren[2], Ellen Sano[2], Jessica E. Biddle[1], Sarah E. Smith-Jeffcoat[1], Melissa A. Rolfes[1], H. Keipp Talbot[8], Carlos G. Grijalva[8], Alexandra M. Mellis[1], Lisa Saiman[2], Raul A. Silverio Francisco[2], Anny L. Diaz Perez[2], Ana M. Valdez de Romero[2], Ayla Bullock[3], Amy Yang[3], Quenla Haehnel[3], Jessica Lin[3], Julienne Reynolds[3], Katherine (Katie) Murray[3], Miriana Moreno Zivanovich[3], Anna McShea[3], Brittney Figueroa[3], Melody Liu[3], Kathleen Grice[4], Cameron Bendalin[4], Sonia Chavez[4], Jolie Granger[4], Ferris Alaa Ramadan[5], Flavia Maria Nakayima Miiro[5], Josue Ortiz[5], Mokenge Ndiva Mongoh[5], Edward A. Belongia[6], Hannah Berger[6], Vicki Moon[6], Gina Burbey[6], Leila Deering[6], Brianna Freund[6], Garrett Heuer[6], Sarah Kopitzke[6], Carrie Marcis[6], Jennifer Meece[6], Jennifer Moran[6], DeeAnn Hertel[6], Joshua Petrie[6], Miriah Rotar[6], Carla Rottscheit[6], Elisha Stefanski[6], Sandy Strey[6], Melissa Strupp[6], Rosita Thiessen[7], Marcela Lopez[7], Alondra A. Aguilar[7], Emma Stainton[7], Grace K-Y. Tam[7], Jonathan Altamirano[7], Leanne X. Chun[7], Rasika Behl[7], Samantha A. Ferguson[7], Yuan J. Carrington[7], Frank S. Zhou[7], Chris Lindsell[8], Judy King[8], John Meghreblian[8], Samuel Massion[8], Brittany Creasman[8], Lauren Milner[8], Andrea Stafford Hintz[8], Jorge Celedonio[8], Ryan Dalforno[8], Maria Catalina Padilla-Azain[8], Daniel Chandler[8], Paige Yates[8], Brianna Schibley-Laird[8], Alexis Perry[8], Ruby Swaimn[8], Mason Speirs[8], Erica Anderson[8], Suryakala Sarilla[8], Amelia Dodds[8], Dayton Marchlewski[8], Timothy Williams[8], Afan Swan[8], Onika Abrams[8], Jackson Resser[8], Ine Sohn[8], Cara Lwin[8], Hsi-nien (Jubilee) Tan[8], Stephen Yeargin[8], James Grindstaff[8], Heather Prigmore[8], Jessica Lai[8], Zhouwen Liu[8], James D. Chappell[8], Marcia Blair[8], Rendie E. McHenry[8], Bryan P. M. Peterson[8], Lauren J. Ezzell[8].
